# Genotype‐specific pathogenic effects in human dilated cardiomyopathy

**DOI:** 10.1113/JP274145

**Published:** 2017-06-01

**Authors:** Ilse A. E. Bollen, Maike Schuldt, Magdalena Harakalova, Aryan Vink, Folkert W. Asselbergs, Jose R. Pinto, Martina Krüger, Diederik W. D. Kuster, Jolanda van der Velden

**Affiliations:** ^1^ Department of Physiology, Amsterdam Cardiovascular Sciences VU University Medical Center Amsterdam the Netherlands; ^2^ Department of Cardiology Division of Heart and Lungs, University of Utrecht University Medical Center Utrecht Utrecht the Netherlands; ^3^ Department of Pathology University Medical Center Utrecht Utrecht the Netherlands; ^4^ Durrer Center for Cardiogenetic Research ICIN–Netherlands Heart Institute Utrecht the Netherlands; ^5^ Institute of Cardiovascular Science, Faculty of Population Health Sciences University College London London UK; ^6^ Department of Biomedical Sciences College of Medicine Florida State University Tallahassee FL USA; ^7^ Institute of Cardiovascular Physiology Heinrich Heine University Düsseldorf Düsseldorf Germany; ^8^ Netherlands Heart Institute Utrecht the Netherlands

**Keywords:** dilated cardiomyopathy, heart failure, protein phosphorylation, troponin

## Abstract

**Key points:**

- Mutations in genes encoding cardiac troponin I (*TNNI3*) and cardiac troponin T (*TNNT2*) caused altered troponin protein stoichiometry in patients with dilated cardiomyopathy.
- *TNNI3_p.98trunc_* resulted in haploinsufficiency, increased Ca^2+^‐sensitivity and reduced length‐dependent activation.
- *TNNT2_p.K217del_* caused increased passive tension.
- A mutation in the gene encoding Lamin A/C (*LMNA* *_p.R331Q_*) led to reduced maximal force development through secondary disease remodelling in patients suffering from dilated cardiomyopathy.
- Our study shows that different gene mutations induce dilated cardiomyopathy via diverse cellular pathways.

**Abstract:**

Dilated cardiomyopathy (DCM) can be caused by mutations in sarcomeric and non‐sarcomeric genes. In this study we defined the pathogenic effects of three DCM‐causing mutations: the sarcomeric mutations in genes encoding cardiac troponin I (*TNNI3_p.98truncation_*) and cardiac troponin T (*TNNT2_p.K217deletion_*; also known as the p.K210del) and the non‐sarcomeric gene mutation encoding lamin A/C (*LMNA_p.R331Q_*). We assessed sarcomeric protein expression and phosphorylation and contractile behaviour in single membrane‐permeabilized cardiomyocytes in human left ventricular heart tissue. Exchange with recombinant troponin complex was used to establish the direct pathogenic effects of the mutations in *TNNI3* and *TNNT2*. The *TNNI3_p.98trunc_* and *TNNT2_p.K217del_* mutation showed reduced expression of troponin I to 39% and 51%, troponin T to 64% and 53%, and troponin C to 73% and 97% of controls, respectively, and altered stoichiometry between the three cardiac troponin subunits. The *TNNI3_p.98trunc_* showed pure haploinsufficiency, increased Ca^2+^‐sensitivity and impaired length‐dependent activation. The *TNNT2_p.K217del_* mutation showed a significant increase in passive tension that was not due to changes in titin isoform composition or phosphorylation. Exchange with wild‐type troponin complex corrected troponin protein levels to 83% of controls in the *TNNI3_p.98trunc_* sample. Moreover, upon exchange all functional deficits in the *TNNI3_p.98trunc_* and *TNNT2_p.K217del_* samples were normalized to control values confirming the pathogenic effects of the troponin mutations. The *LMNA_p.R331Q_* mutation resulted in reduced maximal force development due to disease remodelling. Our study shows that different gene mutations induce DCM via diverse cellular pathways.

## Key points

- Mutations in genes encoding cardiac troponin I (*TNNI3*) and cardiac troponin T (*TNNT2*) caused altered troponin protein stoichiometry in patients with dilated cardiomyopathy.
- *TNNI3_p.98trunc_* resulted in haploinsufficiency, increased Ca^2+^‐sensitivity and reduced length‐dependent activation.
- *TNNT2_p.K217del_* caused increased passive tension.
- A mutation in the gene encoding Lamin A/C (*LMNA* *_p.R331Q_*) led to reduced maximal force development through secondary disease remodelling in patients suffering from dilated cardiomyopathy.
- Our study shows that different gene mutations induce dilated cardiomyopathy via diverse cellular pathways.

**Abbreviations**
cTnC: cardiac troponin C
cTnI: cardiac troponin I
cTnT: cardiac troponin T
DCM: dilated cardiomyopathy
EC_50_: [Ca^2+^] needed to achieve 50% of maximal force
ExAC: Exome Aggregation Consortium
*F*_max_: maximal force
*F*_pass_: passive force
GAPDH: glyceraldehyde 3‐phosphate dehydrogenase
IDCM: idiopathic dilated cardiomyopathy
KO: knock out
LDA: length‐dependent activation
LV: left ventricle
LVAD: left ventricular assist device
PKA: protein kinase A
PKC: protein kinase C
WT: wild‐type

## Introduction

Dilated cardiomyopathy (DCM) is a cardiac disease characterized by dilatation of the left ventricle (LV) and a reduced systolic function. Initially, the prevalence of DCM was determined to be 1:2500 based on phenotypic screening, but recent studies suggested that it could be as high as 1:250 (Hershberger *et al*. 2013). DCM can be caused by environmental factors (viral infection, alcohol abuse, drug toxicity) or have a genetic basis. With current genetic screening, a genetic cause is found in 20–50% of DCM patients (Hershberger *et al*. 2010; Herman *et al*. 2012; van Spaendonck‐Zwarts *et al*. 2013). Over 30 genes have been found to harbour mutations that are likely to cause DCM (Hershberger *et al*. 2013). The Exome Aggregation Consortium (ExAC) recently reported that many rare variants in various sarcomeric and non‐sarcomeric genes, which were assumed to be disease‐causing, only have limited pathogenic burden as no or limited excess variation was found in a DCM population compared with ∼60,000 reference samples (Walsh *et al*. 2017). On the other hand, the presence of rare variants of uncertain significance was reported to be significantly higher in the DCM population compared with the ExAC reference samples indicating an overly conservative estimation of pathogenicity of these variants (Walsh *et al*. 2017). Among the various genes implicated in DCM are genes encoding sarcomeric proteins such as cardiac troponin I (encoded by *TNNI3*) (Carballo *et al*. 2009; van Spaendonck‐Zwarts *et al*. 2013), cardiac troponin T (encoded by *TNNT2*) (Hershberger *et al*. 2009; van Spaendonck‐Zwarts *et al*. 2013; Walsh *et al*. 2017) and titin (encoded by *TTN*) (Herman *et al*. 2012; Walsh *et al*. 2017), and genes encoding for non‐sarcomeric proteins such as lamin A/C (encoded by *LMNA*), a protein involved in nuclear stability (Parks *et al*. 2008; van Spaendonck‐Zwarts *et al*. 2013; Walsh *et al*. 2017). The fact that mutations in proteins of such diverse function can cause DCM implies that multiple pathomechanisms can lead to cardiac dilatation and associated cardiac dysfunction. In this study we defined the pathological effects on cardiomyocyte function of three different DCM‐causing mutations in genes encoding sarcomeric (*TNNI3, TNNT2*) and non‐sarcomeric (*LMNA*) proteins.

**Figure 1:**
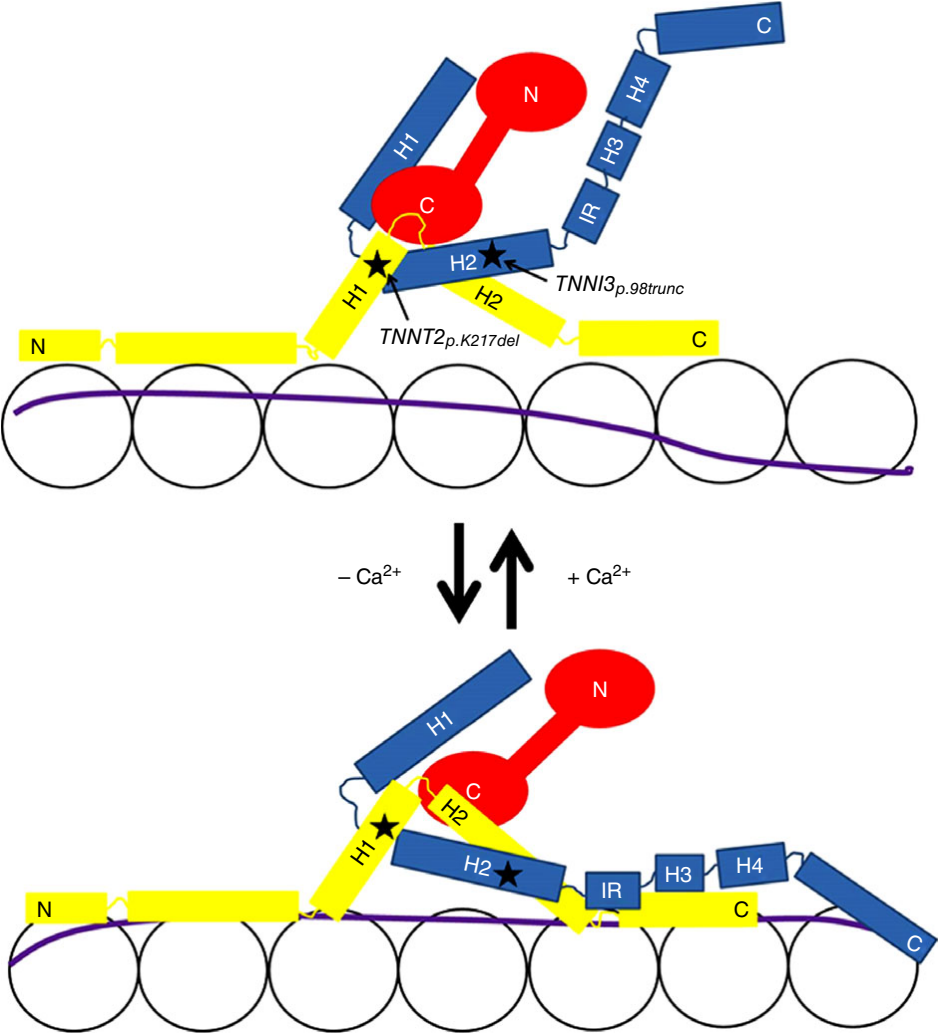
Schematic representation of the troponin complex cTnT is shown in yellow, cTnI in blue and cTnC in red. The letters N and C indicate the N‐ and C‐terminus, respectively. The upper diagram shows the troponin complex in the presence of Ca^2+^ while the lower diagram shows the troponin complex without Ca^2+^. Location of the studied mutations are indicated with stars and an arrow in the upper panel. The letter H indicates a helix structure. IR, inhibitory region.

The troponin complex consists of three different troponin proteins; cardiac troponin T (cTnT), cardiac troponin I (cTnI) and cardiac troponin C (cTnC). The involvement of troponin and tropomyosin in force generation has been described in the three state model of the thin filaments (McKillop & Geeves, 1993). The role of cTnI is to inhibit actin–myosin interaction and, through its interaction with cTnC, plays an important role in the Ca^2+^‐sensitivity of sarcomere activation (Westfall *et al*. 1999). The troponin complex can lock tropomyosin in the blocked state (so called B‐state) at low Ca^2+^ concentrations during which contraction does not occur since tropomyosin sterically hinders the interaction between myosin and actin. During contraction calcium binds to cTnC, which leads to a conformational change that enhances binding of cTnC to cTnI. This results in a large conformational change in cTnI, which leads to displacement of its inhibitory domains away from actin and thereby releasing its inhibitory effect on actin–myosin interaction (Spyracopoulos *et al*. 1997; Stone *et al*. 1998). Tropomyosin moves and transits into the closed state (C‐state), which enables myosin to bind to actin and subsequently cause force generation. The open state (M‐state) is the final shift of tropomyosin after myosin has bound and facilitates the formation of a strong cross‐bridge. By binding to both cTnC and tropomyosin (Li *et al*. 2002), cTnT regulates ATPase activity during contraction, but also serves as an anchor on the thin filaments for the troponin complex. The different troponin proteins as a complex and the location of the mutations studied are shown in a schematic representation in Fig. 1. The 292C→T transition in the *TNNI3* gene encoding cTnI is predicted to result in a premature stop codon at amino acid 98. Truncation in this part of the protein would cause loss of the cTnC and two actin‐binding domains of cTnI (Mogensen *et al*. 2015). The p.K217del (also known as p.K210del; Otten *et al*. 2010) mutation in the *TNNT2* gene has been reported across the world in unrelated families and is associated with high mortality and disease onset at a young age (∼33 years) (Kamisago *et al*. 2000; Mogensen *et al*. 2004; Hershberger *et al*. 2009; Otten *et al*. 2010). Mutations in the non‐sarcomeric gene *LMNA*, encoding the inner nuclear protein lamin A/C, have been found in 6% of DCM patients (Parks *et al*. 2008). Many patients carrying a *LMNA* mutation show conduction abnormalities and arrhythmias (Parks *et al*. 2008; Perrot *et al*. 2009). The *LMNA_p.R331Q_* mutation is located in the coil 2B domain, which is important for homodimerization. The *LMNA_p.R331Q_* mutation is predicted to cause loss of salt‐bridge interaction and thereby affect lamina stability (Gangemi & Degano, 2013).

Apart from the direct mutation‐mediated changes in cardiac function, secondary disease remodelling plays an important role in DCM pathogenesis (Kötter *et al*. 2013; Beqqali *et al*. 2016). Phosphorylation by protein kinase A (PKA) of cTnI can fine‐tune Ca^2+^‐sensitivity and length‐dependent activation (LDA) of sarcomeres (Konhilas *et al*. 2003; Sequeira *et al*. 2013). PKA‐mediated phosphorylation of cTnI upon activation of the β‐adrenergic receptors by adrenaline reduces myofilament Ca^2+^‐sensitivity and enhances LDA (Konhilas *et al*. 2003). In heart failure, the β‐adrenergic receptor system is chronically stimulated leading to down‐regulation and desensitization of the β‐adrenergic receptors and subsequently decreased PKA‐mediated phosphorylation (Harding *et al*. 1994). Therefore, it is important to distinguish between the direct effects of mutations on sarcomere function and the indirect effects through changes in the β‐adrenergic system.

Even though various genes are implicated in DCM, these mutations ultimately result in a dilated heart and cardiac dysfunction. Mutations can induce different cellular changes depending on their effect on protein function. Therefore, patients could have different disease mechanisms leading to DCM. Our studies in human cardiac tissue of DCM patients showed reduced expression of the troponin complex in samples harbouring sarcomeric mutations in *TNNI3_p.98trunc_* and *TNNT2_p.K217del_*. In the *TNNI3_p.98trunc_* sample we did not find a truncated protein, and the haploinsufficiency led to increased Ca^2+^‐sensitivity and reduced LDA. The *TNNT2_p.K217del_* mutation caused increased passive tension (*F*_pass_) and a non‐significant mild reduction in Ca^2+^‐sensitivity. Upon exchange with wild‐type (WT) troponin complex, all parameters normalized to control confirming the pathogenicity of these sarcomeric mutations. The non‐sarcomeric mutation *LMNA_p.R331Q_* showed decreased maximal force (*F*_max_) development and increased Ca^2+^‐sensitivity of sarcomeres, which were both attributed to secondary disease remodelling. Also idiopathic DCM (IDCM) samples showed increased myofilament Ca^2+^‐sensitivity and reduced LDA, which could be attributed to secondary disease remodelling. Therefore, mutations in genes encoding proteins of diverse functions can cause DCM, which implies that changes in different cellular pathways can lead to cardiac dilatation and dysfunction.

## Methods

### Ethical approval

Left ventricular (LV) tissue was obtained from DCM patients who underwent cardiac transplantation, two samples of patients who carried the *LMNA_p.R331Q_* mutation were derived from a biopsy taken prior to LV assist device (LVAD) implantation. The other *LMNA_p.R331Q_* sample was derived from a cardiac transplantation of a heart that had been supported by a LVAD prior to transplantation. Most DCM patient samples used in this study were acquired from the Biobank of the University Medical Centre Utrecht, the Netherlands. This study was approved by the Biobank Research Ethics Committee, University Medical Centre Utrecht, Utrecht, the Netherlands (protocol number WARB 12/387). Written informed consent was obtained. Samples were obtained from regions halfway between the atrioventricular valves and the apex. As control samples we used explanted LV heart tissue of healthy donors – people who had died from a non‐cardiac cause, typically motor vehicle accidents. These healthy donor samples and three DCM were acquired from the University of Sydney, with the ethical approval of the Human Research Ethics Committee no. 2012/2814. The control samples used were 3.160, 4.049, 6.042, 3.162, 5.128, 6.020, 7.044, 3.164, 3.141, 6.008, 5.086, 8.004, 7.054, and the DCM samples used were 4.036, 3.107 and 2.082. All samples were stored in liquid nitrogen or at −80ºC until use.

### Cardiomyocyte force measurements

*F*_max_ and *F*_pass_ of sarcomeres were measured at pCa 4.5 and pCa 9.0, respectively, in single membrane‐permeabilized cardiomyocytes mechanically isolated from heart tissue as previously described (van Dijk *et al*. 2012). LDA experiments and PKA incubations were performed as previously described (van der Velden *et al*. 2000). Ca^2+^‐sensitivity was measured as the [Ca^2+^] needed to achieve 50% of *F*_max_ (EC_50_) and LDA was measured as the shift in EC_50_ (ΔEC_50_) at a sarcomere length of 1.8 μm and 2.2 μm.

### Protein expression and phosphorylation

#### Titin

Titin isoforms were separated on a 1% (w/v) agarose gel and stained with SYPRO Ruby protein stain (Invitrogen, Carlsbad, CA, USA) as described previously (Warren *et al*. 2003) and samples were measured in triplicate. Phosphorylation of titin was assessed as previously described (Kötter *et al*. 2016). For titin phosphorylation, site‐specific antibodies directed to Ser4010 (N2Bunique sequence (N2Bus) domain; PKA and extracellular signal‐regulated kinase 2 (ERK2) target), and Ser12022 and Ser11878 (PEVK domain; protein kinase C (PKC) and Ca^2+^/calmodulin‐dependent protein kinase II (CaMKIIδ) target) were used.

#### Troponin

The troponin proteins were separated by 12% polyacrylamide and 4–15% precast gradient gels (BioRAD, Hercules, CA, USA) gel electrophoresis and Western blots were stained with specific antibodies (cTnI: Abcam, Cambridge, UK, ab10231; cTnT: Sigma, St. Louis, MO, USA, T6277; cTnC: Santa Cruz, Dallas, TX, USA, sc48347) to determine their expression, which was corrected by expression of other cellular proteins (glyceraldehyde 3‐phosphate dehydrogenase (GAPDH): Cell signaling, 2118S, Cell signaling, Danvers, MA, USA; α‐actinin: Sigma, A7811). The *TNNI3_p98.trunc_* and *TNNT2_p.K217del_* samples were measured in duplicate of which the average is shown. Phosphorylation of cTnI was assessed as previously described (Zaremba *et al*. 2007).

### Troponin exchange

The troponin complex was exchanged in membrane‐permeabilized cardiomyocytes as previously described (Wijnker *et al*. 2013). The recombinant WT or *TNNT2_p.K217del_* troponin complex was added to the cells in a concentration of 1 mg ml^−1^. The recombinant troponin complexes could be distinguished from highly phosphorylated endogenous troponin since the recombinant troponins were not phosphorylated. Quantification of the exchange rate was performed by phos‐tag analysis in which non‐, mono‐ and bis‐phosphorylated cTnI (Pierce, Rockford IL, USA, MA1‐22700) were separated by polyacrylamide‐bound Mn^2+^‐phos‐tag gel electrophoresis Western blotting as previously described (Najafi *et al*. 2015). The percentage of recombinant troponin complex present after exchange was quantified as the percentage of non‐phosphorylated cTnI to the total of non‐, mono‐ and bis‐phosphorylated cTnI levels. Total cTnI levels after exchange were quantified by cTnI (Abcam, ab10231) and corrected for myosin light chain‐2 (MLC2) (Enzo, Farmingdale, NY, USA, ALX‐BC‐1150).

### Statistics

Graphpad Prism software was used for statistical analysis. *F*_max_ of DCM cardiomyocytes compared to control cardiomyocytes was compared with one‐way ANOVA and Tukey's *post hoc* test. LDA was calculated as ΔEC_50_. Ca^2+^‐sensitivity and passive tension in DCM cardiomyocytes were compared to control cardiomyocytes by two‐way ANOVA. All values are shown as mean ± standard error of the mean. A *P* value <0.05 was considered to represent a significant difference and is indicated with an asterisk in figures. The 95% confidence intervals (CI) of the control group are indicated with a dotted line in the graphs and was used to assess the difference of the sample of interest compared to controls in situations where only a single data point of the sample of interest could be used.

## Results

### Diverse functional myofilament changes in DCM with sarcomeric and non‐sarcomeric mutations

**Figure 2:**
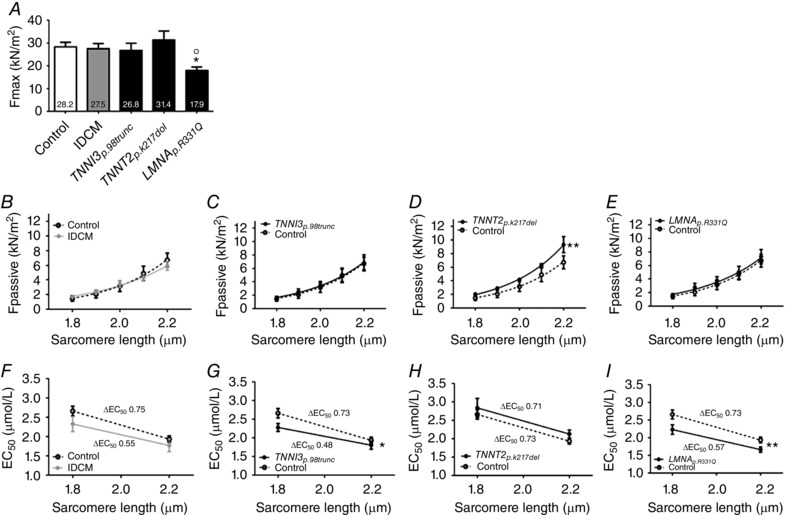
Baseline contractile properties *A*, *F*_max_, measured at pCa 4.5, was significantly decreased (*P* < 0.05) in *LMNA_p.R331Q_* samples (17.9 ± 1.6 kN m^−2^, *N* = 3, *n* = 19) compared to controls (28.2 ± 2.1 kN m^−2^, *N* = 6, *n* = 21) while IDCM (27.5 ± 2.3 kN m^−2^, *N* = 5, *n* = 18), *TNNI3_p.98trunc_* (26.8 ± 3.2 kN m^−2^, *N* = 1, *n* = 13) and *TNNT2_p.K217del_* (31.4 ± 3.9 kN m^−2^, *N* = 1, *n* = 14) samples showed similar *F*_max_ as controls. Data obtained from Hoorntje *et al*. (2016). *B*, *F*_pass_, measured at pCa 9.0, in membrane‐permeabilized cardiomyocytes of IDCM (*N* = 5, *n* = 19), were similar compared to control (*N* = 4, *n* = 10). *C*, *F*_pass_, measured at pCa 9.0, in membrane‐permeabilized cardiomyocytes of *TNNI3_p.98trunc_* sample (*N* = 1, *n* = 6), were similar compared to control (*N* = 4, *n* = 10). *D*, *F*_pass_, measured at pCa 9.0, in membrane‐permeabilized cardiomyocytes of *TNNT2_p.K217del_* patient (*N* = 1, *n* = 8), was significantly increased (*P* < 0.01) compared to control (*N* = 4, *n* = 10). *E*, *F*_pass_, measured at pCa 9.0, in *LMNA_p.R331Q_* samples (*N* = 3, *n* = 12), were similar compared to control (*N* = 4, *n* = 10). *F*, Ca^2+^‐sensitivity was non‐significantly increased and ΔEC_50_ was non‐significantly reduced in IDCM (*N* = 5, *n* = 11) compared to control (*N* = 6, *n* = 13). *G*, Ca^2+^‐sensitivity was significantly increased (*P* < 0.05) in *TNNI3_p.98trunc_* patient (*N* = 1, *n* = 7) compared to control (*N* = 6, *n* = 13), ΔEC_50_ was non‐significantly reduced. *H*, Ca^2+^‐sensitivity was only slightly and non‐significantly reduced compared to controls and ΔEC_50_ was preserved in *TNNT2_p.K217del_* sample (*N* = 1, *n* = 7) compared to control (*N* = 6, *n* = 13). *I*, Ca^2+^‐sensitivity was significantly increased (*P* < 0.01) in *LMNA_p.R331Q_* samples (*N* = 3, *n* = 7) compared to control (*N* = 6, *n* = 13) while ΔEC_50_ was preserved. N, number of samples; n, number of total cardiomyocytes measured.

The 12 control samples used for experiments consisted of four females and eight males with a mean age of 44.5 ± 3.4 years. The patient with *TNNI3_p.98trunc_* mutation was a 46‐year‐old male and the patient with *TNNT2_p.K217del_* mutation was a 19‐year‐old male. Three patients with *LMNA_p.R331Q_* were studied of which two were male and one female with a mean age of 45.3 ± 3.4 years. The IDCM samples consisted of four males and one female with a mean age of 54.6 ± 3.2 years. To determine the functional properties of human DCM samples with sarcomeric and non‐sarcomeric protein mutations, sarcomere function was measured in single isolated membrane‐permeabilized cardiomyocytes at various [Ca^2+^] to assess passive and active properties of the sarcomeres. No difference in *F*_max_ (Fig. 2 *A*) was observed in IDCM, *TNNI3_p.98trunc_* and *TNNT2_p.K217del_* cardiomyocytes, while *F*_max_ was significantly lower in cardiomyocytes with the *LMNA_p.R331Q_* mutation (17.9 kN m^−2^, data from Hoorntje *et al*. 2016) compared to controls (28.2 kN m^−2^). A previous study showed that this decreased *F*_max_ was due to decreased myofibril density (Hoorntje *et al*. 2016). *F*_pass_ is an important determinant of diastolic function which was measured at low [Ca^2+^] (pCa 9.0) over a range of sarcomere lengths (Granzier & Irving, 1995). IDCM samples (Fig. 2 *B*), *TNNI3_p.98trunc_* (Fig. 2 *C*) and *LMNA_p.R331Q_* (Fig. 2 *E*) showed a comparable *F*_pass_ development over the range of sarcomere lengths compared to controls. The *TNNT2_p.K217del_* cardiomyocytes showed a significant increase in *F*_pass_ compared to controls, which was most pronounced at longer sarcomere lengths (Fig. 2 *D*). In addition to changes in *F*_pass_, stretching of cardiomyocytes during filling of the heart increases active force development. This LDA of myofilaments is the cellular basis of the Frank–Starling mechanism (Sequeira *et al*. 2013; Beqqali *et al*. 2016). We measured active force development over a range of [Ca^2+^] at sarcomere lengths 1.8 μm and 2.2 μm to study LDA. IDCM samples showed an increased Ca^2+^‐sensitivity and reduced LDA compared to controls (Fig. 2 *F*). The *TNNI3_p.98trunc_* cardiomyocytes showed Ca^2+^‐sensitivity was increased and LDA was blunted (Fig. 2 *G*) compared to controls. *TNNT2_p.K217del_* cardiomyocytes showed only a minor and non‐significant decrease in Ca^2+^‐sensitivity and LDA was preserved (Fig. 2 *H*). The Ca^2+^‐sensitivity of *LMNA_p.R331Q_* cardiomyocytes was significantly increased compared to controls, while LDA was preserved (Fig. 2 *I*). Overall these data illustrate that changes in passive and active myofilament properties differ between different sarcomeric gene mutations and between sarcomeric and non‐sarcomeric mutations.

### Haploinsufficiency and altered stoichiometry of troponin proteins in DCM with *TNNI3_p.98trunc_* and *TNNT2_p.K217del_* mutations

**Figure 3:**
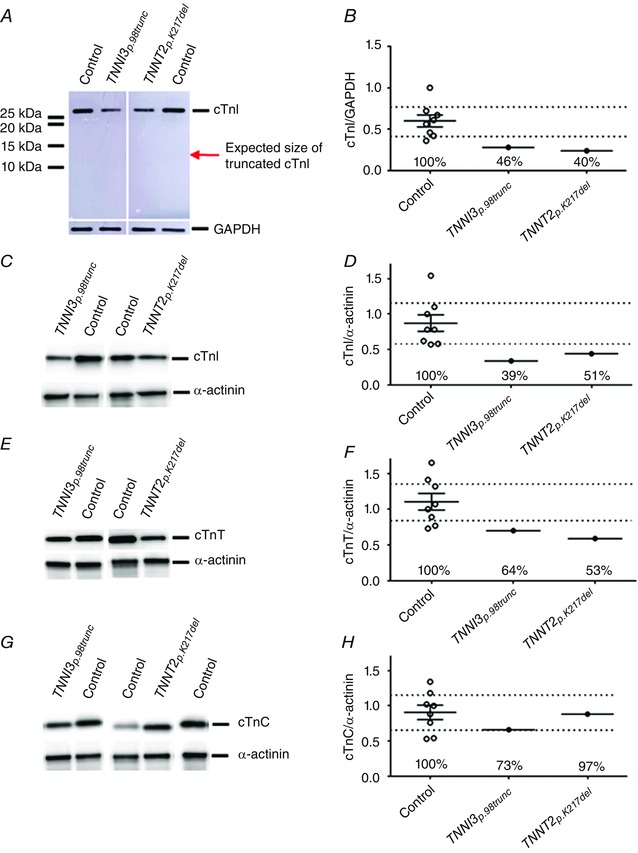
Expression of troponin in troponin mutants *A* and *B*, cTnI levels measured with an antibody directed to the N‐terminal of cTnI and normalized to GAPDH were decreased to 46% in the *TNNI3_p.98trunc_* (0.28) and to 40% in *TNNT2_p.K217del_* (0.24) samples compared to controls (*N* = 8, mean = 0.60, CI = 0.43–0.77). *A*, corresponding gel image showed no additional bands indicative of a truncated cTnI protein. *C* and *D*, cTnI levels were decreased to 39% in *TNNI3_p.98trunc_* (0.34) and to 51% in *TNNT2_p.K217del_* (0.44) samples compared to controls (*N* = 8, mean = 0.87, CI = 0.59–1.15) when normalized for α‐actinin. *E* and *F*, cTnT levels normalized to α‐actinin were also decreased to 64% in *TNNI3_p.98trunc_* (0.70) and to 53% in *TNNT2_p.K217del_* (0.59) samples compared to controls (*N* = 8, mean = 1.11, CI = 0.83–1.38). *G* and *H*, cTnC levels normalized to α‐actinin were slightly decreased to 73% in *TNNI3_p.98trunc_* sample (0.66) but still within the 95% CI of controls (*N* = 8, mean = 0.91, CI = 0.67–1.15). *TNNT2_p.K217del_* showed normal (0.88, 97% of controls) cTnC levels. [Color figure can be viewed at wileyonlinelibrary.com]

Since the troponin complex and the interactions between the various troponin proteins are important for adequate contractile behaviour of the myofilaments, we studied the composition of the troponin complex in the *TNNI3_p.98trunc_* and *TNNT2_p.K217del_* samples. We observed that cTnI protein level was decreased in both the *TNNI3_p.98trunc_* and *TNNT2_p.K217del_* samples compared to controls when normalized to the cytoplasmic housekeeping protein GAPDH (Fig. 3 *A* and *B*). If the truncated cTnI protein is present in the *TNNI3_p.98trunc_* sample an antibody raised against the N‐terminus of cTnI is expected to show two bands: the native protein and the truncated protein. However, only one band was visible at the height of the native cTnI protein (Fig. 3 *A*). This indicates that the mutant protein is either not expressed or efficiently degraded resulting in cTnI haploinsufficiency. The level of cTnI relative to the sarcomeric housekeeping gene α‐actinin was also decreased in both *TNNI3_p.98trunc_* and *TNNT2_p.K217del_* samples (Fig. 3 *C* and *D*), which indicates that less cTnI is present within the sarcomeres itself. The reduction in cTnI levels was accompanied by a less pronounced decrease in cTnT (Fig. 3 *E* and *F*) and near normal cTnC levels (Fig. 3 *G* and *H*). This indicates that the *TNNI3_p.98trunc_* and *TNNT2_p.K217del_* samples have altered stoichiometry of the three troponin proteins since the decrease in cTnI, cTnT and cTnC is not to the same extent.

### Hypophosphorylation of cTnI underlies increased Ca^2+^‐sensitivity in DCM with the *LMNA_p.R331Q_* mutation, but does not underlie myofilament defects in DCM with *TNNI3_p.98trunc_* and *TNNT2_p.K217del_* mutations

**Figure 4:**
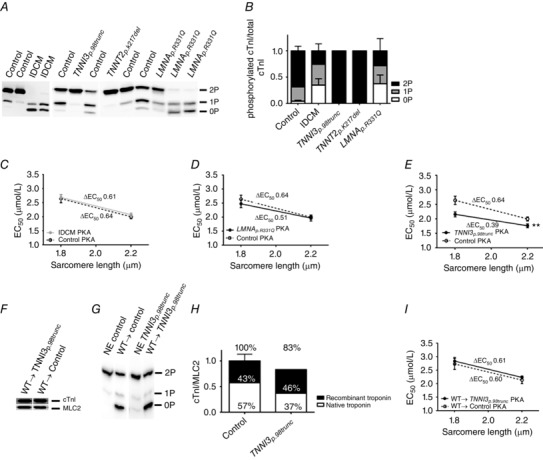
Secondary disease remodelling and direct mutation effects *A*, phos‐tag analysis showed separation of non‐ (0P), mono‐ (1P) and bis‐ (2P) phosphorylated cTnI. *B*, phosphorylation of cTnI was increased in *TNNI3_p.98trunc_* and *TNNT2_p.K217del_* samples compared to controls (*N* = 7) while cTnI phosphorylation in *LMNA_p.R331Q_* (*N* = 3) and IDCM (*N* = 3) was decreased compared to controls. *C*, Ca^2+^‐sensitivity was normalized in IDCM cardiomyocytes (*N* = 5, *n* = 12) compared to control cardiomyocytes (*N* = 6, *n* = 14) after incubation with exogenous PKA. *D*, Ca^2+^‐sensitivity was normalized in *LMNA_p.R331Q_* cardiomyocytes (*N* = 3, *n* = 7) compared to control cardiomyocytes (*N* = 6, *n* = 14) after incubation with exogenous PKA. *E*, after incubation with exogenous PKA, Ca^2+^‐sensitivity in *TNNI3_p.98trunc_* cardiomyocytes (*N* = 1, *n* = 7) remained significantly increased (*P* < 0.01) compared to control cardiomyocytes (*N* = 6, *n* = 14). *F*, exchange with WT troponin complex restored cTnI levels in the *TNNI3_p.98trunc_* sample to 83% of that of controls exchanged with WT troponin complex (*H*). *G*, phos‐tag gel analysis showed high phosphorylation of native troponin complex prior to exchange (NE) and incorporation of unphosphorylated recombinant protein after exchange. *H*, the 83% was composed of 46% recombinant troponin and 37% native troponin in the *TNNI3_p.98trunc_* sample compared with 43% recombinant troponin in the control exchanged with WT troponin complex. *I*, Ca^2+^‐sensitivity and LDA were restored in *TNNI3_p.98trunc_* cardiomyocytes (*N* = 1, *n* = 9) compared to control (*N* = 2, *n* = 11) after exchange with WT troponin complex and incubation with exogenous PKA. N, number of samples; n, number of total cardiomyocytes measured.

The troponin complex was reduced in both the *TNNI3_p.98trunc_* and *TNNT2_p.K217del_* samples, while cardiomyocytes from these samples showed different myofilament properties. We therefore studied phosphorylation of cTnI using phos‐tag analysis. Control samples showed prominent bis‐ and mono‐phosphorylated cTnI and low non‐phosphorylated cTnI (Fig. 4 *A* and *B*). IDCM samples showed reduced cTnI phosphorylation as is evident by prominent non‐ and mono‐phosphorylated cTnI bands and a weak bis‐phosphorylated cTnI band (Fig. 4 *A* and *B*). In the *TNNI3_p.98trunc_* and *TNNT2_p.K217del_* samples cTnI was highly phosphorylated indicated by an intense bis‐phosphorylated cTnI band and very weak non‐ and mono‐phosphorylated cTnI bands (Fig. 4 *A* and *B*). On the contrary, the *LMNA_p.R331Q_* samples showed decreased cTnI phosphorylation evident from prominent non‐ and mono‐phosphorylated cTnI bands (Fig. 4 *A* and *B*). Low phosphorylation of cTnI has been reported previously in DCM (Wijnker *et al*. 2014) and may underlie increased Ca^2+^‐sensitivity (Konhilas *et al*. 2003; Wijnker *et al*. 2014) and a blunted LDA (Konhilas *et al*. 2003; Wijnker *et al*. 2014). Indeed, after incubation with exogenous PKA we observed normalization of Ca^2+^‐sensitivity to controls in IDCM (Fig. 4 *C*) and in the *LMNA_p.R331Q_* cardiomyocytes (Fig. 4 *D*). Incubation with exogenous PKA did not change Ca^2+^‐sensitivity in controls. Also LDA was restored in IDCM samples compared to controls after incubation with exogenous PKA (Fig. 4 *C*). However, Ca^2+^‐sensitivity was still significantly increased compared to controls after incubation with exogenous PKA in the *TNNI3_p.98trunc_* cardiomyocytes (Fig. 4 *E*). These experiments confirm that impaired β‐adrenergic receptor signalling, and subsequent hypophosphorylation of cTnI, is the cause of the increased Ca^2+^‐sensitivity in IDCM and the *LMNA_p.R331Q_* cardiomyocytes, while the observed increased Ca^2+^‐sensitivity and impaired LDA is a direct mutation effect in the *TNNI3_p.98trunc_* cardiomyocytes.

### Correction of high Ca^2+^‐sensitivity and blunted LDA in DCM with *TNNI3_p.98trunc_* by human recombinant WT troponin

Next we aimed to assess whether the observed haploinsufficiency of cTnI, and reduced cTnT and cTnC, caused increased Ca^2+^‐sensitivity and reduced LDA in the *TNNI3_p.98trunc_* cardiomyocytes. We exchanged the endogenous troponin complex with the recombinant WT troponin complex in order to restore total troponin levels. The level of cTnI increased to 83% after exchange (Fig. 4 *F* and *H*), relative to the cTnI level in control cells exchanged with exogenous recombinant WT troponin complex. The 83% cTnI in the *TNNI3_p.98trunc_* sample after exchange consisted of 46% recombinant troponin complex and 37% native troponin complex, as determined by phos‐tag gel analysis (Fig. 4 *G* and *H*). In the control sample 43% of total cTnI levels was derived from the recombinant troponin complex (Fig. 4 *G* and *H*). This indicates that we exchanged endogenous troponin complex with recombinant WT complex, but also added additional recombinant WT troponin complex in the exchange process thereby largely overcoming the haploinsufficiency in the *TNNI3_p.98trunc_* cardiomyocytes. Since the recombinant troponin complex is unphosphorylated we incubated the exchanged cells with exogenous PKA prior to functional cell measurements. Upon exchange with the WT troponin complex both Ca^2+^‐sensitivity as well as LDA were normalized to control values in the *TNNI3_p.98trunc_* cardiomyocytes (Fig. 4 *I*).

### High passive force in DCM with *TNNT2_p.K217del_* is caused by the mutation and not by changes in isoform composition or phosphorylation of titin

**Figure 5:**
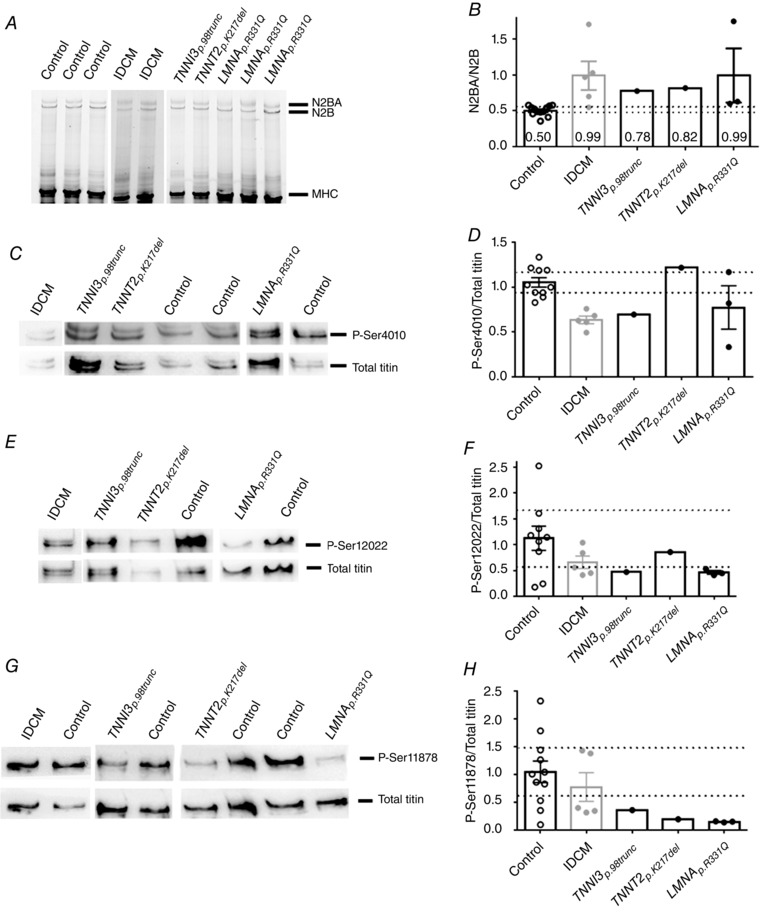
Alterations in titin isoform composition and phosphorylation in DCM mutants *A*, titin isoforms, N2BA and N2B, separated by agarose gel electrophoresis. *B*, titin N2BA/N2B ratios were increased in IDCM (0.99 ± 0.20, *N* = 5), *TNNI3_p.98trunc_* (0.78, *N* = 1), *TNNT2_p.K217del_* (0.82, *N* = 1) and *LMNA_p.R331Q_* (0.99 ± 0.38, *N* = 3) samples compared to controls (0.50 ± 0.02, *N* = 12, CI = 0.49–0.55). *C*, phosphorylated Ser4010 compared to total titin levels. *D*, titin phosphorylation at Ser4010 was decreased in IDCM (*N* = 5), *TNNI3_p.98trunc_* (*N* = 1) and *LMNA_p.R331Q_* (*N* = 3) compared to controls (*N* = 10, CI = 0.93–1.17), while *TNNT2_p.K217del_* (*N* = 1) showed slight increased phosphorylation of Ser4010 compared to control. *E*, phosphorylated Ser12022 compared to total titin levels. *F*, titin phosphorylation at Ser12022 was decreased in *TNNI3_p.98trunc_* (*N* = 1), *TNNT2_p.K217del_* (*N* = 1) and *LMNA_p.R331Q_* (*N* = 3), compared to control (*N* = 9, CI = 0.58–1.66), while phosphorylation at Ser12022 was within the 95% CI of controls in IDCM (*N* = 5). *G*, phosphorylated Ser11878 compared to total titin levels. *H*, titin phosphorylation at Ser11878 was decreased in *TNNI3_p.98trunc_* (*N* = 1), *TNNT2_p.K217del_* (*N* = 1) and *LMNA_p.R331Q_* (*N* = 3), compared to control (*N* = 11, CI = 0.62–1.49) while phosphorylation at Ser11878 was within the 95% CI of controls in IDCM (*N* = 5).

**Figure 6:**
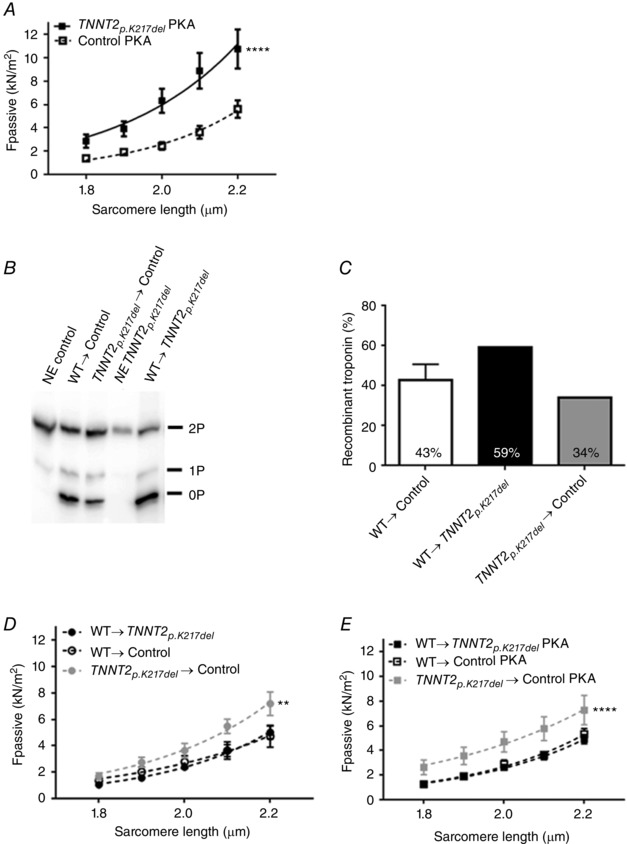
*TNNT2_p.K217del_* increases passive tension *A*, *F*_pass_ remained significantly increased (*P* < 0.0001) in *TNNT2_p.K217del_* cardiomyocytes (*N* = 1, *n* = 9) compared to controls (*N* = 4, *n* = 13) after incubation with exogenous PKA. *B*, phos‐tag gel analysis showed high phosphorylation of native troponin complex prior to exchange (NE) and incorporation of unphosphorylated recombinant protein after exchange. *C*, after exchange 43% of present troponin complex in controls was recombinant WT cTnI while in the *TNNT2_p.K217del_* sample this was 59% and in control exchanged with *TNNT2_p.K217del_* mutant troponin complex this was 34%. *D*, upon exchange with WT troponin complex, cardiomyocytes of *TNNT2_p.K217del_* (*N* = 1, *n* = 11) showed restoration of *F*_pass_ compared to controls exchanged with WT troponin complex (*N* = 2, *n* = 8) while *F*_pass_ was significantly increased (*P* = 0.001) in control cardiomyocytes exchanged with mutant *TNNT2_p.K217del_* troponin complex (*N* = 2, *n* = 7). *E*, after incubation with exogenous PKA, cardiomyocytes of *TNNT2_p.K217del_* exchanged with recombinant WT troponin complex (*N* = 1, *n* = 13) showed normalization of *F*_pass_ compared to control cardiomyocytes exchanged with WT troponin complex (*N* = 2, *n* = 9) while *F*_pass_ was significantly increased (*P* < 0.0001) in control cardiomyocytes exchanged with mutant *TNNT2_p.K217del_* troponin complex (*N* = 2, *n* = 7). N, number of samples; n, number of total cardiomyocytes measured.

We next set out to determine the cause of the increased *F*_pass_ in the *TNNT2_p.K217del_* sample. An important determinant of *F*_pass_ is titin isoform composition (Makarenko *et al*. 2004; Nagueh *et al*. 2004). Titin can exist as a stiff isoform (N2B) or a larger, more compliant isoform (N2BA). All DCM groups showed an increase in compliant titin compared to controls independent of the type of mutation (Fig. 5 *A* and *B*). The observed increase in N2BA/N2B ratio cannot explain the high *F*_pass_ in *TNNT2_p.K217del_*. Therefore, we examined phosphorylation of titin at three well‐established phosphorylation sites in the elastic I‐band region. Phosphorylation of Ser4010 on titin, a target of PKA, is known to decrease *F*_pass_ (Kötter *et al*. 2013), while PKC‐mediated phosphorylation of Ser12022 and Ser11878 results in increased *F*_pass_ (Hidalgo *et al*. 2009). While phosphorylation at Ser4010 was lower in the IDCM, *TNNI3_p.98trunc_* and *LMNA_p.R331Q_* samples compared to controls, a preserved or even slightly increased Ser4010 phosphorylation was observed in the *TNNT2_p.K217del_* sample (Fig. 5 *C* and *D*). Ser12022 (Fig. 5 *E* and *F*) and Ser11878 (Fig. 5 *G* and *H*) phosphorylation, which would increase *F*_pass_, was within the 95% CI of controls in IDCM but lower in samples carrying mutations compared to controls. Therefore, the increase in *F*_pass_ in the *TNNT2_p.K217del_* cardiomyocytes is not caused by alterations in titin phosphorylation at the investigated sites. The increase in *F*_pass_ was not due to impaired PKA‐mediated phosphorylation of titin since *F*_pass_ remained significantly higher in *TNNT2_p.K217del_* cardiomyocytes compared to controls after incubation with exogenous PKA (Fig. 6 *A*). Exchange with the WT troponin complex led to a 59% incorporation of recombinant troponin complex in the *TNNT2_p.K217del_* sample (Fig. 6 *B* and *C*). The exchange normalized *F*_pass_ in the *TNNT2_p.K217del_* cardiomyocytes to control level (Fig. 6 *D*). In addition, after exchange of the mutant *TNNT2_p.K217del_* troponin complex into a healthy control sample we observed that only 34% of total troponin present after exchange was recombinant (Fig. 6 *B* and *C*). However, this was sufficient to cause a significant increase in *F*_pass_ (Fig. 6 *D*). The increase in *F*_pass_ upon exchange with *TNNT2_p.K217del_* in a control sample was not due to impaired PKA‐mediated phosphorylation since after incubation with exogenous PKA *F*_pass_ remained significantly increased (Fig. 6 *E*). These results indicate that the *TNNT2_p.K217del_* mutant protein itself increases *F*_pass_.

## Discussion

Mutations in various sarcomeric and non‐sarcomeric genes can induce DCM. In this study we aimed to define the pathogenic effects of the sarcomeric *TNNI3_p98.trunc_* and *TNNT2_p.K217del_* mutations, and the non‐sarcomeric *LMNA_p.R331Q_* mutation. Our study provides proof that the two sarcomere mutations cause myofilament dysfunction, while changes in myofilament properties in IDCM and the non‐sarcomeric mutation samples are the result of secondary disease remodelling. One of the *LMNA_p.R331Q_* samples showed a smaller decrease in cTnI phosphorylation compared to the other two samples. This was the sample obtained from a patient who used a LVAD prior to transplantation. It is therefore possible that the LVAD has partly reversed the secondary remodelling (Sakamuri *et al*. 2016). However, this patient did not show deviations from the other two *LMNA_p.R331Q_* patients in other protein analyses.

### Haploinsufficiency and altered stoichiometry of troponin proteins in human DCM

We show that the *TNNI3_p.98trunc_* sample does not lead to a truncated protein but causes haploinsufficiency. This is in line with Kostareva *et al*. who showed that a truncation in the *TNNI3* gene at the 176th amino acid in a patient with restrictive cardiomyopathy did not lead to a truncated protein, but instead to a 50% reduction of cTnI (Kostareva *et al*. 2009). The decrease in cTnI in our patient was associated with decreased levels of cTnT, while cTnC levels remained near normal. Also in the *TNNT2_p.K217del_* sample reduced levels of cTnI and cTnT were observed albeit to a smaller extent than observed in the *TNNI3_p.98trunc_* sample. While cTnI was most reduced in both samples, this was accompanied by a less pronounced decrease in cTnT, while cTnC levels remained near normal leading to altered stoichiometry. Since cTnT itself can bind to the thin filaments through tropomyosin and serves as an anchor for the whole troponin complex, this might explain why we observed a smaller decrease in cTnT protein levels compared to cTnI, which is more dependent on the formation of the whole troponin complex to attach to the thin filaments. This is in line with a study by Feng *et al*. in which they showed that an expression level of 25% of cTnI was accompanied by a 53% decrease in cTnT (Feng *et al*. 2009).

### A *TNNI3* truncation mutation in human DCM causes haploinsufficiency, high Ca^2+^‐sensitivity and impaired LDA of myofilaments

Various mutations in *TNNI3* have been shown to increase Ca^2+^‐sensitivity and subsequently impair cardiac relaxation leading to hypertrophic cardiomyopathy (HCM) (Takahashi‐Yanaga *et al*. 2001). These observations have been attributed to the possibility that the mutations act as a poison peptide and ‘lock’ tropomyosin in the C‐ or M‐state. They are suggested to increase the stability of the Ca^2+^‐bound form of the thin filaments or destabilize the Ca^2+^‐free form of the thin filaments (Kobayashi & Solaro, 2006). In this study, we show that the *TNNI3_p.98trunc_* mutation in DCM patient cardiomyocytes also increased Ca^2+^‐sensitivity and in addition impaired LDA, which could not be corrected with exogenous PKA (Figs 2 *G* and 4 *E*) while the increased Ca^2+^‐sensitivity and impaired LDA in IDCM could be corrected by exogenous PKA (Figs 2 *F* and 4 *C*). This is in line with Sequeira *et al*. who reported that the HCM‐causing *TNNI3_p.R145W_* mutation impaired LDA, which could not be rescued with exogenous PKA (Sequeira *et al*. 2013). However, it has been heavily debated if mutations in *TNNI3* and *TNNT2* are able to cause DCM or HCM through haploinsufficiency. Homozygous *TNNT2* knock out (KO) mice are embryonically lethal, while heterozygous *TNNT2* KO mice have reduced cTnT mRNA levels, but normal cTnT protein levels, and show no cardiac phenotype (Ahmad *et al*. 2008). The *TNNT2* gene apparently has robust compensatory mechanisms in order to maintain protein levels. In addition, full *TNNI3* KO in mice is lethal around 18 days of age (Liu *et al*. 2007; Feng *et al*. 2009), while heterozygous *TNNI3* KO mice survive without detectable phenotype (Feng *et al*. 2009). Feng *et al*. suggested that a cTnI threshold of 25% WT protein exists for the mice to survive. They also showed that cTnI is likely to be produced in excess amounts under healthy conditions (Feng *et al*. 2009). *TNNI3* KO mice were characterized by impaired diastolic function as an early cardiac phenotype, followed by enlarged cardiac dimensions and overt heart failure (Liu *et al*. 2007; Feng *et al*. 2009). In support of impaired diastolic dysfunction, an increased resting tension in isolated ventricular myocytes of *TNNI3* KO mice has been found (Huang *et al*. 1999). However, we did not find any alterations in *F*_pass_ in the *TNNI3_p.98trunc_* cardiomyocytes. In the early postnatal life of *TNNI3* KO mice, slow skeletal TnI (ssTnI) production was maintained in order to compensate for the absence of cTnI (Huang *et al*. 1999; Liu *et al*. 2007; Feng *et al*. 2009). Although ssTnI was elevated in KO mice for a longer period than in WT littermates, it also decreased over time and the compensatory effect was gradually lost. Ca^2+^‐sensitivity decreased in *TNNI3* KO mice along with the decrease of ssTnI. However, compared to WT littermates of the same age, the *TNNI3* KO mice showed an increased Ca^2+^‐sensitivity (Huang *et al*. 1999). This is in line with the increased Ca^2+^‐sensitivity we observed in the *TNNI3_p.98trunc_* cardiomyocytes, and with the increased Ca^2+^‐sensitivity of ATPase activity in rabbit skeletal muscle upon extraction of TnI that has been reported previously (Shiraishi & Yamamoto, 1994). Using troponin exchange experiments in single human cardiomyocytes, we were able to increase cTnI levels close to control levels and normalize Ca^2+^‐sensitivity and LDA in the *TNNI3_p.98trunc_* cardiomyocytes. Our data prove that the increased Ca^2+^‐sensitivity and impaired LDA were directly caused by the mutation‐induced haploinsufficiency.

### Secondary disease‐related changes in DCM with sarcomeric and non‐sarcomeric mutations

In line with previous reports in human DCM (Makarenko *et al*. 2004; Nagueh *et al*. 2004; Beqqali *et al*. 2016), all DCM patients showed an increase in compliant titin, indicated by a higher N2BA/N2B ratio compared to controls (Fig. 5 *A* and *B*). The increase in compliant titin therefore seems to be a general hallmark of DCM and not a specific effect of the mutations studied. Despite the increase in compliant titin, *F*_pass_ was similar to controls in IDCM, the *LMNA_p.R331Q_* and *TNNI3_p.98trunc_* cardiomyocytes. Interestingly, PKA‐mediated phosphorylation titin was unaltered in the *TNNT2_p.K217del_* sample. In addition, cTnI phosphorylation was also not affected in the *TNNT2_p.K217del_* and *TNNI3_p.98trunc_* samples suggesting that these specific mutations do not lead to defects in β‐adrenergic receptor signalling. This is contrary to what we observed in the IDCM samples and to what has been reported in other DCM samples (Wijnker *et al*. 2014) and might indicate that mutations in troponin can impair phosphorylation through local signalling.

### The *TNNT2_p.K217del_* mutation causes high passive stiffness in human cardiomyocytes

The *TNNT2_p.K217del_* cardiomyocytes showed increased *F*_pass_ (Fig. 2 *D*). Since the troponin levels in the *TNNI3_p.98trunc_* and *TNNT2_p.K217del_* sample were reduced in a similar fashion we expect that the poison peptide of *TNNT2_p.K217del_* and not reduced troponin complex was the cause of the high *F*_pass_. We hypothesize that the mutant *TNNT2_p.K217del_* troponin complex is less likely to incorporate in the sarcomeres than the WT troponin complex. Also the observed decreased cTnT levels in *TNNT2_p.K217del_* might indicate the mutant protein is not as stable as healthy cTnT. We observed a low exchange rate of the mutant *TNNT2_p.K217del_* protein complex in a control sample (34%) and a high exchange rate of the WT troponin complex in the *TNNT2_p.K217del_* sample (59%) compared to the exchange rate of WT in a control sample (43%) (Fig. 6 *C*). Most models have high incorporation of the mutant with values reported to be 79% (Michael *et al*. 2016) and an estimated incorporation of ∼55% (Morimoto *et al*. 2002). The limited incorporation of the mutant cTnT in our study in combination with the decrease in total troponin levels we observed have important implications. The mutant protein levels are probably higher in exchange experiments in healthy tissue, knock in (KI) or transgenic mouse models than in human patients. In addition, total troponin levels might not be affected in these models while we show they can be decreased in human patient tissue. Therefore, cardiomyocytes of DCM patients with the *TNNT2_p.K217del_* mutation might have different contractile performance than reported in previously published animal models. In support of this, a transgenic mouse model of the *TNNT2_p.K217del_* mutation showed that the severity of DCM is related to the ratio of mutant *vs*. WT transcript (Ahmad *et al*. 2008). Inoue *et al*. also showed an increase in *F*_pass_ in a mouse KI model of this mutation (Inoue *et al*. 2013). They indicated that part of the *F*_pass_ increase was titin based, but they did not find an increase in N2B titin. Inoue *et al*. proposed that the increase in *F*_pass_ might be due to increased PKC‐mediated phosphorylation of titin although they did not assess titin phosphorylation. We observed an increase in compliant titin and lower PKC‐mediated phosphorylation in the *TNNT2_p.K217del_* sample (Fig. 5), neither of which could explain the high *F*_pass_. Our troponin exchange experiments provided proof that the *TNNT2_p.K217del_* mutation itself causes a significant increase in *F*_pass_, irrespective of PKA‐mediated phosphorylation. To our knowledge we are the first to show that the *TNNT2_p.K217del_* mutation causes a profound increase in *F*_pass_ in human heart tissue. The lysine at 217 is part of the H1 helix of cTnT which directly interacts with tropomyosin. The interaction between cTnT and tropomyosin might therefore be affected by the *TNNT2_p.K217del_* mutation. The *TNNT2_p.K217del_* might cause tropomyosin to be available for residual cross‐bridge interaction even at low calcium concentrations resulting in high *F*_pass_. We observed a mild, though non‐significant decrease in Ca^2+^‐sensitivity in *TNNT2_p.K217del_* cardiomyocytes compared to controls. The lysine at 217 in cTnT is believed to be involved in calcium‐sensitive cTnC binding (Tanokura *et al*. 1983). Decreased Ca^2+^‐sensitivity has been reported in various studies that exchanged human WT or *TNNT2_p.K217del_* in various animal models (Morimoto *et al*. 2002; Venkatraman *et al*. 2003; Michael *et al*. 2016), while another study showed no effect on Ca^2+^‐sensitivity (Bai *et al*. 2013). In addition, a KI mouse model (Du *et al*. 2007; Inoue *et al*. 2013; Memo *et al*. 2013) and a heterozygous KO mouse model with transgenic expression of *TNNT2_p.K217del_* also showed decreased Ca^2+^‐sensitivity (Ahmad *et al*. 2008). An impaired interaction of cTnT with cTnI and cTnC due to the *TNNT2_p.K217del_* mutation has been reported (Mogensen *et al*. 2004), while another study showed no significant difference in the secondary structure of *TNNT2_p.K217del_* measured as α‐helical content (Venkatraman *et al*. 2003). We hypothesize that we only observed a minor decrease in Ca^2+^‐sensitivity in the *TNNT2_p.K217del_* cardiomyocytes due to the reduced level of total troponin complex. In the *TNNI3_p.98trunc_* sample we observed an increased Ca^2+^‐sensitivity, which was corrected upon troponin exchange which increased troponin complex levels. Decreased troponin complex levels combined with the Ca^2+^‐desensitizing effect of the *TNNT2_p.K217del_* mutation might have counteracted each other leading to a negligible effect on myofilament Ca^2+^‐sensitivity. Reports about *F*_max_ in *TNNT2_p.K217del_* mutants range from no effect (Morimoto *et al*. 2002; Du *et al*. 2007; Ahmad *et al*. 2008; Inoue *et al*. 2013; Michael *et al*. 2016) to a decrease (Venkatraman *et al*. 2003; Bai *et al*. 2013). In our study we did not find a decrease in *F*_max_ in the *TNNT2_p.K217del_* sample (Fig. 2 *A*). Inoue *et al*. also showed a depressed Frank–Starling mechanism (Inoue *et al*. 2013) which we could not confirm in the human patient tissue and was also not observed in a transgenic mouse model of *TNNT2_p.K217del_* (Ahmad *et al*. 2008). A KI mouse model, transgenic mouse model, or exchange experiments might give rise to different levels of mutant protein in the sarcomeres and explain the different results on force‐generating capacity. Differences in the ability of the body to degrade the mutant protein and to compensate with the healthy allele might cause variable penetrance, age of onset and severity in human patients.

### Conclusion

**Figure 7:**
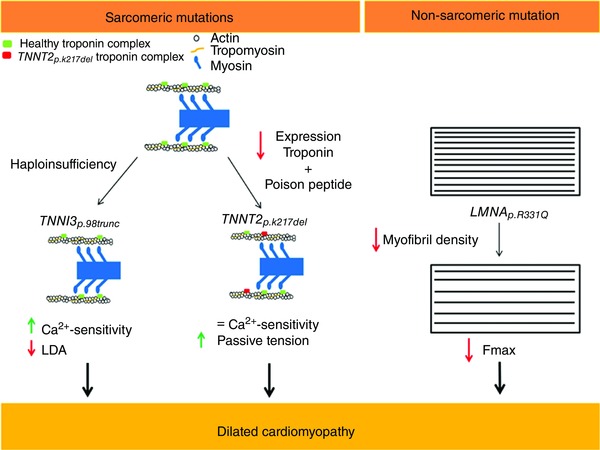
Overview of pathogenic effects of *TNNI3_p.98trunc_*, *TNNT2_p.K217del_* and *LMNA_p.R331Q_* The *TNNI3_p.98trunc_* mutation did not result in a truncated protein and instead caused haploinsufficiency leading to increased Ca^2+^‐sensitivity and impaired LDA. The *TNNT2_p.K217del_* mutation might act as a poison peptide and caused decreased Ca^2+^‐sensitivity as shown by others. We showed that the sample with *TNNT2_p.K217del_* mutation resulted in decreased expression of the troponin proteins and in addition has a poison peptide effect. Since the decreased expression of the troponin proteins increased Ca^2+^‐sensitivity and the poison peptide decreased Ca^2+^‐sensitivity, there was no significant change in Ca^2+^‐sensitivity in the *TNNT2_p.K217del_* sample. In addition, *F*_pass_ was increased. The *LMNA_p.R331Q_* mutation caused decreased myofibril density and subsequent impaired contractility.

Mutations in different sarcomeric and non‐sarcomeric genes lead to DCM and we have shown that these mutations trigger different pathological routes leading to end‐stage dilated hearts (Fig. 7). In this study we show that the sarcomeric mutations *TNNI3_p.98trunc_* and *TNNT2_p.K217del_* cause reduced expression of the troponin complex and altered stoichiometry between the troponin subunits. In the *TNNI3_p.98trunc_* cardiomyocytes this led to increased Ca^2+^‐sensitivity, which could not be corrected with exogenous PKA but was normalized to control levels upon exchange with WT troponin complex. The *TNNT2_p.K217del_* mutation caused a mild, non‐significant, reduction in Ca^2+^‐sensitivity and significantly increased *F*_pass_, which could not be corrected by PKA but was normalized to control levels upon exchange with WT troponin complex. In addition, incorporation of the *TNNT2_p.K217del_* mutant troponin complex in a control sample confirmed the mutant protein itself causes increased *F*_pass._ This implies that even mutations in the genes encoding for the troponin proteins have different effects on myofilament function. In contrast, the *LMNA_p.R331Q_* mutation caused reduced maximal force development and increased Ca^2+^‐sensitivity due to secondary disease remodelling. Also IDCM samples showed an increased Ca^2+^‐sensitivity due to secondary disease remodelling. We show that although DCM patients present general hallmarks, the causative mutations underlie different cellular changes. Based on our studies, we propose that different mutations cause DCM via diverse pathways.

## Additional information

### Competing interests

None declared.

### Author contributions

I.A.E.B., D.W.D.K. and J.V.D.V. conceived, designed and coordinated the study and wrote the paper. I.A.E.B. created Figs 1 and 7 and performed and analysed the experiments shown in Figs 2, 4, 5 and 6. M.S. performed and analysed the experiments shown in Figs 4 and 6. M.H., A.V. and F.W.A. were involved in patient data and material acquisition. J.R.P. created recombinant protein complexes used in Figs 4 and 6. M.K. provided antibodies and supervision for experiments in Fig. 5. Experiments shown in Figs 2, 3, 4, 5A,B and 6 were performed at the Department of Physiology at the VU University Medical Center in Amsterdam, the Netherlands. Experiments shown in Fig 5 C‐H were performed at the Insititute for Cardiovascular Physiology at the Heinrich‐Heine University in Düsseldorf, Germany. All authors critically revised the manuscript, reviewed the results and approved the final version of the manuscript. All authors agree to be accountable for all aspects of the work. All persons designated as authors qualify for authorship, and all those who qualify for authorship are listed.

### Funding

We acknowledge the support from the Netherlands Cardiovascular Research Initiative, an initiative with the support of the Dutch Heart Foundation, CVON2011‐11 ARENA and CVON2014‐40 DOSIS, and Rembrandt Institute for Cardiovascular Sciences 2013. J.R.P. is supported by the National Heart, Lung and Blood Institute of the National Institutes of Health (Grant HL128683). F.W.A. is supported by a Dekker scholarship (Junior Staff Member 2014T001) of the Dutch Heart Foundation and UCL Hospitals NIHR Biomedical Research Centre.
